# Elucidation of Phytochemical Content of *Cupressus macrocarpa* Leaves: In Vitro and In Vivo Antibacterial Effect against Methicillin-Resistant *Staphylococcus aureus* Clinical Isolates

**DOI:** 10.3390/antibiotics10080890

**Published:** 2021-07-22

**Authors:** Nashwah G. M. Attallah, Walaa A. Negm, Engy Elekhnawy, Elshaymaa I. Elmongy, Najla Altwaijry, Hala El-Haroun, Thanaa A. El-Masry, Suzy A. El-Sherbeni

**Affiliations:** 1 Pharmaceutical Sciences Department, College of Pharmacy, Princess Nourah Bint Abdulrahman University, Riyadh 84428, Saudi Arabia; ngmohamed@pnu.edu.sa (N.G.M.A.); naaltwaijry@pnu.edu.sa (N.A.); 2 Egyptian Drug Authority (EDA), Giza 8655, Egypt (previously NODCAR); 3 Pharmacognosy Department, Faculty of Pharmacy, Tanta University, Tanta 31111, Egypt; walaa.negm@pharm.tanta.edu.eg (W.A.N.); suzy.elsherbini@pharm.tanta.edu.eg (S.A.E.-S.); 4 Pharmaceutical Microbiology Department, Faculty of Pharmacy, Tanta University, Tanta 31111, Egypt; 5 Pharmaceutical Chemistry Department, Faculty of Pharmacy, Helwan University, Helwan 11795, Egypt; 6 Histology Department, Faculty of Medicine, Menoufia University, Shibin Al Kawm 32511, Egypt; elharoun@yahoo.com; 7 Pharmacology Department, Faculty of Pharmacy, Tanta University, Tanta 31111, Egypt; thanaa.elmasri@pharm.tanta.edu.eg

**Keywords:** trichrome stain, membrane depolarization, *mec*A gene, qRT-PCR, SEM

## Abstract

Methicillin-resistant *Staphylococcus aureus* (MRSA) is an important human pathogen that causes various infections. The increasing resistance of MRSA to different antibiotics is widely spreading; therefore, plant extracts may be novel therapeutic alternatives. The phytochemical profiling of *Cupressus macrocarpa* Hartw. ex Gordon leaves in vitro, and in vivo, antimicrobial potential of its extracts against MRSA clinical isolates were explored. A phytochemical tentative identification of 49 compounds was performed in the leaves using LC-ESI-MS/MS; in addition, isolation, and structure elucidation of hesperidin and eriocitrin were achieved for the first time. The diethyl ether extract (DEEL) exhibited the best antibacterial effect with MIC values ranging from 2 to 8 µg/mL, which significantly reduced the growth and efflux activity in 48.78% and 29.26% of isolates, respectively. qRT-PCR showed a significant down expression of *nor*A and *nor*B genes, which significantly affected the bacterial cell morphology and had a non-significant effect on membrane depolarization (using flow cytometry). In a rat model, four groups were wounded and treated with normal saline or DEEL, or infected with MRSA, or infected and treated with DEEL. The regeneration of the epidermis, maturation of granulation tissue, and reduction of inflammatory cell infiltration were observed after treatment with DEEL. Thus, *C. macrocarpa* leaves may be a promising source for new antimicrobials against MRSA.

## 1. Introduction

*Cupressus macrocarpa* Hartw. ex Gordon *(Callitropsis macrocarpa*) or Monterey cypress (family Cupressaceae) [1,2] is a well-known ornamental tree. It is a gymnosperm plant widely distributed in the United States of America, and warm temperate and subtropical regions in Europe, New Zealand, North Africa, and Asia. It is commonly used as an ornament and for windbreaks [3]. It was used traditionally for decades to treat different diseases and ailments, e.g., whooping cough, as a styptic, to eliminate fluid retention, and rheumatism [4].

*C. macrocarpa* leaves and branchlets are rich in essential oils [5,6] composed mainly of monoterpenes and traces of sesquiterpenes [7] as well as diterpenes [8], flavonoids, biflavonoids [4,9], and other compounds. The diethyl ether extract is composed mainly of essential oil and non-volatile components, e.g., diterpenes and diterpenoids [10]. It is reported that the essential oils possess a powerful antimicrobial effect [6] and, according to the literature [11], the Essential Oils (Eos) of *C. macrocarpa* have an antifungal effect against *Trichophyton rubrum*, a specific dermal fungus. This was the motive to extensively study the antibacterial effect of *C. macrocarpa*.

Flavonoids are bioactive common components of plants with diverse and remarkable biological activities such as antioxidant or free radical scavenging, anti-inflammatory [12], cardioprotective [13], anticarcinogenic [14], antimicrobial [15], and antiviral [16] activities.

They have a basic C_15_ phenyl-benzopyrone nucleus, which is biosynthesized from shikimic and acetic or malonic acid precursors. Their structures earn their diversity through differing numbers and positions of substituents including hydroxyl, methoxyl, and glycosyl groups. Glycosides may be of *O*-, *C*-, or *O*- and *C*-types. Biflavonoids are dimers of flavonoids, usually flavones and flavanones. The interflavonoid linkage is either a C-C bond or occasionally an ether bond. The prevalent C-C attached types are: 6,8″-linked (agathisflavone group), 8,8″-linked (cupressuflavone group), 3′,8″-linked (amentoflavone group) and others. These compounds are restricted in distribution, to be found prevalently in Gymnosperms [17].

Phytochemical investigation of flavonoids, biflavonoids, and other compounds for *C. macrocarpa* leaves was not previously studied. In this study, chemical profiling was achieved by LC-ESI-MS/MS technique, which supplied us with valuable data about this plant.

The nontargeting small molecule comprehensive analysis of large-scale plant metabolomics that depend on liquid chromatography coupled with electrospray ionization tandem mass spectrometry (LC-ESI-MS/MS) is considered a ubiquitous technique for recognition of different natural products in plants as it can enrich us with information about molecular mass, molecular fragmentation, molecular formula, the relative abundance of different components, as well as determining the type of glycosides and position of substituents.

*Staphylococcus aureus* is an important human pathogenic bacterium that can cause various infections ranging from superficial skin infections to fatal systemic infections [18]. *S. aureus* is considered one of the leading causes of nosocomial infections worldwide [19]. The misuse of penicillin has resulted in the emergence of penicillin-resistant *S. aureus*. In addition, the more problematic bacteria, methicillin-resistant *S. aureus* (MRSA), have started to spread around the world, causing high rates of morbidity and mortality [20]. This health care problem necessitates the exploring for novel, natural compounds with an antibacterial effect against MRSA isolates. Plants are usually used traditionally for the treatment of certain diseases; thus, many researchers have focused on exploring the antibacterial activity of different plants [21].

This study was conducted to investigate the phytochemical constituents of *C. macrocarpa* leaves by LC-ESI-MS/MS analysis of the methanol extract of defatted leaves. To the best of our knowledge, there are no reports concerning the antibacterial impact of *C. macrocarpa* leaf extracts against MRSA isolates. Therefore, an inspection of the potential in vitro and in vivo antibacterial effect of *C. macrocarpa* leaf extracts against MRSA isolates was carried out.

## 2. Results

### 2.1. Results of LC-ESI-MS/MS Analysis of C. Macrocarpa Leaves Methanol Extract

In the current study, the data-independent acquisition-based identification by mass spectral deconvolution using MS-DIAL in LC-ESI-MS/MS analysis (negative mode ESI) of *C. macrocarpa* leaves methanol extract revealed the tentative identification of 49 compounds of phenolic acids, flavones, flavonols, flavanones, isoflavones, biflavonoids, catechin, stilbene glycosides, and diterpenes. Flavonoids aglycones, *O*- and *C*-glycosides were identified. Results are listed in Table 1. The total ion chromatogram (TIC) of methanol extract of *C. macrocarpa* leaves (negative mode) is presented in Supplementary Materials Figure S1. Whereas, the structures of different compounds are demonstrated in Supplementary Materials Figures S2 and S3.

#### 2.1.1. Characterization of Flavonoids Aglycones

Recognition of different flavonoids aglycons was conducted by an inhouse database to identify luteolin, acacetin, 3, 5, 7-trihydroxy-4′-methoxy flavone, myricetin, quercetin, 3′-methyl quercetin (3′-methoxy-4′,5,7-trihydroxyflavonol), hesperetin, and naringenin through their pseudomolecular ions at *m/z* 285.038, 283.090, 299.055, 317.055, 301.036, 315.169, 301.118, and 271.064, respectively.

#### 2.1.2. Characterization of Flavones Glycosides and a Biflavone

The common neutral loss of 46 Daltons was observed in MS^2^ fragmentations of flavones glycoside due to loss of CO_2_ + H_2_. Luteolin-7-*O*-glucoside exhibited [M−H]¯ ions at *m/z* 447.096. The neutral loss of 162 Daltons of hexose or glucose moiety was detected at *m/z* 285.040. The pseudo-molecular ions of Luteolin-3′,7-di-*O*-glucoside at *m/z* 609.146, with loss of neutral ions of two molecules of hexose or glucose (324 Da), was observed by fragment ion of aglycone at *m/z* 285.201. Apigenin-7-neohesperidoside (rhoifolin) showed pseudo-molecular ions at *m/z* 577.156, neutral loss of CO_2_ (44 Da) indicated at *m/z* 532.902, and loss of sugar part (308 Da) at *m/z* 269.104. The pseudo-molecular ions and the aglycone fragment ions of apigenin-7-*O*-glucoside were detected at *m/z* 431.092 and 269.035, respectively. The pseudo-molecular ions of baicalein-7-*O*- glucuronide (baicalin) were noted at *m/z* 445.169 and the baicalein ions fragment showed at *m/z* 269.142 due to loss of glucuronide moiety. Cupressuflavone or 8,8″bi-apigenin showed pseudo-molecular ions at *m/z* 537.021, which were identified according to the reported data [22].

#### 2.1.3. Characterization of Flavonol Glycosides

Quercitrin pseudo-molecular ions demonstrated at *m/z* 447.180 with loss of rhamnose moiety (146 Da) showing at *m/z* 301.151 and characteristic fragments at *m/z* 179.070 and 151.046. The same loss was recorded for quercetin-7-*O*-rhamnoside. The [M−H]¯ ions were noted at *m/z* 433.077 for quercetin-3-*O*-arabinoside with fragment ions at *m/z* 389.175 for loss of CO_2_ and *m/z* 301.037 for loss of pentose moiety (132 Da). The same loss was observed for quercetin-4′-*O*-glucoside and quercetin-3-*O*-xyloside with [M−H]¯ ions at *m/z* 463.119 and 433.080, respectively, as well as common fragments ions at *m/z* 301 after the loss of hexose or glucose (162 Da) and pentose or xylose (132 Da). The pseudo-molecular ions of quercetin-3,4′-*O*-*β*-diglucopyranoside were at *m/z* 625.071, and loss of di-hexose or di-glucose moieties (324 Da), which was noticed due to the existence of mass fragment ions of quercetin at *m/z* 301.012. The pseudo-molecular ions and kaempferol ion fragments of kaempferol-3-*O*-glucuronide were found at *m/z* 461.127 and 285.103, respectively. Kaempferol-3-*O*-α-L-arabinoside exerted the [M−H]¯ ions at *m/z* 417.134 with loss of 132 Daltons of pentose or arabinose fragments at *m/z* 285.92, the [M−H]¯ ions of kaempferol-3-*O*-α-L-rhamnoside and myricetin-3-*O*-rhamnoside (myricitrin) were exhibited at *m/z* 431.192 and 463.171, respectively. The neutral loss of deoxy hexose or rhamnose was found at *m/z* 285.213 and 317.103, respectively. Ion fragments of RDA (retro-Diels-Alder) at *m/z* 125.023 and 119.035 showed in MS^2^ of kaempferol-3-*O*-α-L-rhamnoside. Syringetin-3-*O*-glucoside and syringetin-3-*O*-galactoside exerted their pseudo-molecular ions at *m/z* 507.102 and 507.112, respectively, and the loss of neutral ions of hexose (glucose or galactose) moiety (162 Da) was observed at *m/z* 345.057 and 345.124, respectively. Isorhamnetin-3-*O*-glucoside showed [M−H]¯ ions at *m/z* 477.098 with fragment ions of isorhamnetin at *m*/*z* 315.041, isorhamnetin-3-*O*-rutinoside exhibited [M-H]¯ ions at *m/z* 623.208, neutral loss of CO_2_ (44 Da) at *m/z* 579.156, and loss of rutinoside (308 Da) at *m/z* 315.036.2.1.4. Characterization of flavanone glycosides

The pseudo-molecular ions of naringenin-7-*O*-glucoside and eriodictyol-7-*O*-glucoside were observed at *m/z* 433.115 and 449.102, respectively, as well as their aglycones at *m/z* 271.053 and 287.032, respectively. The [M−H]¯ ions at *m/z* 595.286 and the aglycone ion fragments at *m/z* 287.030 of eriodictyol-7-*O*-rutinoside were observed, and eriodictyol was formed after neutral loss of rutinoside (308 Da). Hesperidin showed pseudo-molecular ions at *m/z* 609.144 and fragment ions at *m/z* 301.027 for hesperetin.

#### 2.1.4. Characterization of Isoflavone

The [M−H]¯ ions at *m/z* 415.159 were recognized for daidzein-8-*C*-glucoside with neutral loss of hexose or glucose (162 Da) at *m/z* 253.165.

#### 2.1.5. Characterization of Phenolic Compounds

The [M−H]¯ ions at *m/z* 115.081, 173.044, 191.045, 163.039, 289.067, 183.034, 611.117, 181.049, and 359.176 corresponded to maleic, shikimic, quinic, p-coumaric acids, catechin, 3,4-dihydroxy mandelate, neohesperidin dihydrochalcone, syringaldehyde, and rosmarinic acids, respectively. Shikimic and quinic acids showed fragment ions at *m/z* 155.060 and 173.046 due to neutral loss of water. They also shared common ions at *m/z* 109 and 93.

#### 2.1.6. Characterization of Other Compounds

Different compounds of 1-*O-β*-D-glucopyranosyl sinapate, phlorizin, okanin-4′-*O*-glucoside, E-3,4,5′-trihydroxy-3′-glucopyranosyl-stilbene, and syringaldehyde exhibited *m/z* at 385.184, 435.084, 449.114, and 405.093, respectively. Diterpenoids as isocupressic acid, acetylisocupressic acid, and agathadiol were established through the reported mass spectrometry data [23].

### 2.2. Structure Elucidation of Compounds Isolated from C. Macrocarpa Leaves

#### 2.2.1. Structure Identification of Hesperidin

^1^H NMR (DMSO-d_6_, 500 MHz) δ_H_: 5.52 (H-2; 1H, dd, *J* = 12.0, 3.5 Hz), 2.55 (H-3axile; 1H, dd, *J* = 16.0, 4.0 Hz), 3.78 (H-3equatorial; 1H, dd, *J* = 16.0, 12.0 Hz), 6. 12 (H-6; 1H, d, *J* = 2.5 Hz), 6.14 (H-8; 1H, d, *J* = 2.5 Hz), 6.96 (H-2′; 1H, d, *J* = 2.0 Hz), 6.91 (H-5′; 1H, d, *J* = 8.5 Hz), 6.94 (H-6′; 1H, dd, *J* = 2.0, 8.5 Hz), 3.80 (4′- OMe; 3H, s), 9.12 (3′-OH; 1H, s), 12.01 (5-OH; 1H, s), Glucose moiety: 4.97 (H-1″; 1H, d, *J* = 7.5 Hz), 3.20-3.60 (H-2″ to H-6″, m), Rhamnose moiety: 4.69 (H-1′′′; 1H, d, *J* = 3.0 Hz), 3.20-3.60 (H-2‴ to H-5‴, m), 1.09 (6′’’; 3H, d, *J* = 6.0). ^13^C NMR(DMSO-d_6_, 125 MHz)δ: 78.45 (C-2), 40.89 (C-3), 197.48 (C-4), 163.28 (C-5), 96.82 (C-6), 165.57 (C-7), 95.82 (C-8), 162.51 (C-9), 103.56 (C-10), 131.28 (C-1′), 114.61 (C-2′), 146.93 (C-3′), 148.43 (C-4′), 114.61 (C-5′), 118.39 (C-6′), 56.15 (O-Me). Glucose moiety (C-1″ to 6″): 99.91, 74.5, 72.53, 70.13, 76.68, 65.95. Rhamnose moiety (C-1‴ to 6‴): 101.07, 68.84, 70.68, 76.04, 71.28, 18.28. ESI-MS *m/z* 609.144 [M−H]^−^.

#### 2.2.2. Structure Elucidation of Eriocitrin

**Figure 1:**
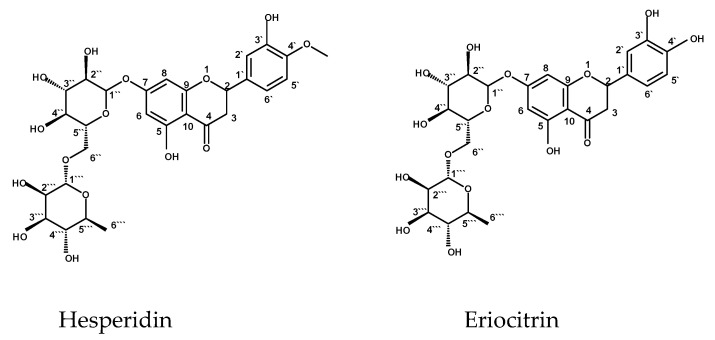
Isolated compounds from *C. macrocarpa* leaves.

^1^H NMR (DMSO-d_6_, 500 MHz) δ_H_: 5.21 (H-2; 1H, dd, *J* = 12.5, 3.5 Hz), 2.88 (H-3axile; 1H, dd, *J* = 17.0, 3.5 Hz), 3.09 (H-3equatorial; 1H, dd, *J* = 17.0, 12.5 Hz), 6. 09 (H-6; 1H, d, *J* = 2.5 Hz), 6.11 (H-8; 1H, d, *J* = 2.5 Hz), 6.92 (H-2′; 1H, d, *J* = 1.5 Hz), 6.60 (H-5′; 1H, d, *J* = 8.5 Hz), 6.89 (H-6′; 1H, dd, *J* = 1.5, 8.5 Hz), Glucose moiety: 4.94 (H-1″; 1H, d, *J* = 7.5 Hz), 3.35-4.25 (H-2″ to H-6″, m), Rhamnose moiety: 4.88 (H-1‴; 1H, d, *J* = 3.0 Hz), 3.35-4.25 (H-2‴ to H-5‴, m), 1.11 (6‴; 3H, d, *J* = 6.0). ^13^C NMR (DMSO-d_6_, 125 MHz) δ: 80.9 (C-2), 44.89 (C-3), 197.23 (C-4), 163.14 (C-5), 98.12 (C-6), 165.27 (C-7), 96.88 (C-8), 162.59 (C-9), 104.16 (C-10), 130.98 (C-1′), 112.11 (C-2′), 146.53 (C-3′), 148.13 (C-4′), 114.32 (C-5′), 118.13 (C-6′). Glucose moiety (C-1″ to 6″): 101.12, 74.90, 73.09, 71.70, 77.11, 66.80. Rhamnose moiety (C-1‴ to 6‴): 102.11, 70.32, 72.08, 76.81, 72.40, 18.01. ESI-MS *m/z* 595.286 [M−H]^−^. Figure 1 represents structures of hesperidin and eriocitrin.

### 2.3. Results of In Vitro Antibacterial Activity of C. Macrocarpa Leaves Extracts

The total methanol extract (TMEL), diethyl ether extract of leaves (DEEL), and methanol extract of defatted leaves (MEDL) exhibited antibacterial effects on the tested MRSA isolates using the agar well-diffusion method. Their minimum inhibitory concentration (MIC) values were detected using the broth microdilution method. The MIC values for the DEEL of *C. macrocarpa* ranged from 2 to 8 µg/mL (the most potent). However, the MIC values for MEDL and TMEL ranged from 256 to 1024 µg/mL and 1024 to 2048 µg/mL, respectively. The MIC values for the positive control (vancomycin) ranged from 0.5 to 4 µg/mL.

#### 2.3.1. Growth Curve Assay

**Figure 2:**
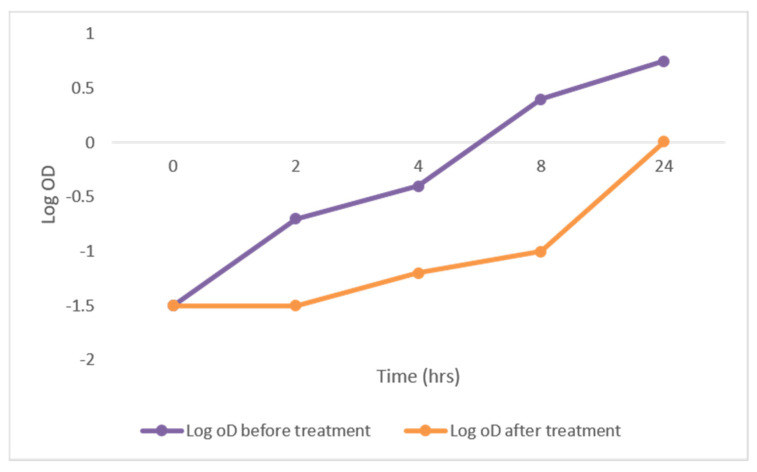
The growth curve of representative MRSA isolates showing a significant reduction in growth after treatment with DEEL.

To determine the effect of DEEL on the growth of MRSA, the growth curves of the tested isolates were analyzed before and after treatment. A significant reduction of 48.78% in the growth of the tested isolates was observed. A representative example is shown in Figure 2.

#### 2.3.2. Efflux Assay

The efflux pump activity is an important mechanism for antibiotic resistance. Herein, the efflux activity of MRSA isolates was determined by evaluating the ability of each isolate to pump out ethidium bromide (EtBr) out of the bacterial cell. The efflux activity of the isolates was categorized into negative, intermediate, and positive using the EtBr cartwheel method. As shown in Table 2, out of 41 MRSA isolates, 12 (29.26%) isolates showed a decrease in their efflux pump activity after treatment with DEEL (i.e., their efflux pump activity was converted from positive to intermediate or negative).

#### 2.3.3. Quantitative RT-PCR

**Figure 3:**
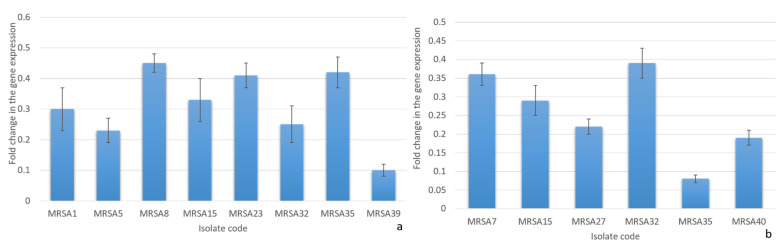
The charts show the fold changes in the transcriptional levels of (**a**) *nor*A and (**b**) *nor*B genes after treatment with the diethyl ether extract of *C. macrocarpa* leaves.

Quantitative reverse transcription-polymerase chain reaction (qRT-PCR) was utilized to study the effect of DEEL on the efflux activity of MRSA isolates (*n* = 12), which exhibited a decrease in their efflux activity by the cartwheel method. The transcriptional levels of the tested efflux pump genes *nor*A and *nor*B were remarkably decreased after treatment, with mean values of fold changes ranging from 0.1 to 0.45 (in 66.66% of clinical isolates) and from 0.08 to 0.39 (in 50% of clinical isolates), respectively, as shown in Figure 3. The expression level of the *nor*C gene showed nonsignificant fold change after treatment.

#### 2.3.4. Membrane Depolarization Assay

**Figure 4:**
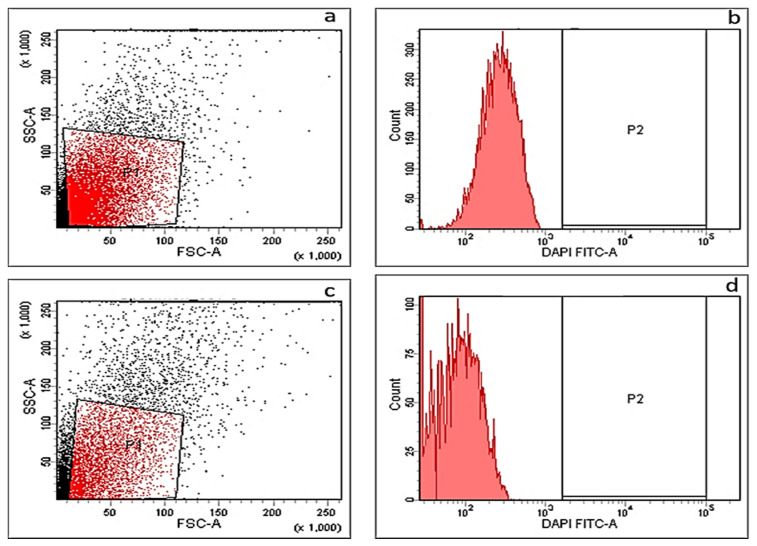
A representative example for MRSA isolates showing nonsignificant change in membrane depolarization detected by flow cytometer. Dot plot (**a**) and histogram (**b**) before treatment (showing a fluorescent gap of 52.6%), dot plot (**c**) and histogram (**d**) after treatment (showing a fluorescent gap of 51.3%).

Membrane depolarization was measured before and after treatment with DEEL using DiBAC4(3), which is a fluorescent indicator dye that can enter the cells when depolarized; thus, membrane depolarization can be studied by recording the fluorescence of this dye using a flow cytometer. We observed nonsignificant change in membrane depolarization after treatment. A representative example is shown in Figure 4.

#### 2.3.5. Examination of Bacterial Cell Morphology by Scanning Electron Microscope (SEM)

**Figure 5:**
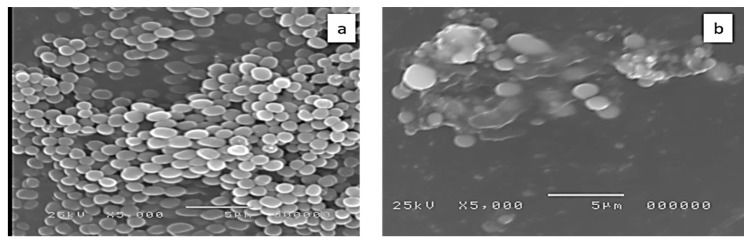
Scanning electron microscope image of a representative MRSA isolate (**a**) before treatment and (**b**) after treatment with the diethyl ether extract of *C. macrocarpa* leaves.

Examination of the cell morphology of MRSA isolates by SEM was carried out to study the ultrastructural changes that occurred after treatment with DEEL. By comparing the treated cells with the untreated ones, we observed that the treated cells were shrinking and there were some degradations of the cell walls, as shown in Figure 5.

#### 2.3.6. Cytotoxicity Assay

The cytotoxic effect of *C. macrocarpa* DEEL in a human skin fibroblast (HSF) cell line was determined using SRB (Sulforhodamine B) assay [24]. The results revealed that IC_50_ of *C. macrocarpa* DEEL against HSF cell line was 21.3 ± 3.41 µg/mL in comparison with doxorubicin as a positive control (IC_50_ = 4.36 ± 0.52 µg/mL).

### 2.4. In Vivo Antibacterial Activity Testing

#### 2.4.1. Wound Closure Percentage (%)

**Figure 6:**
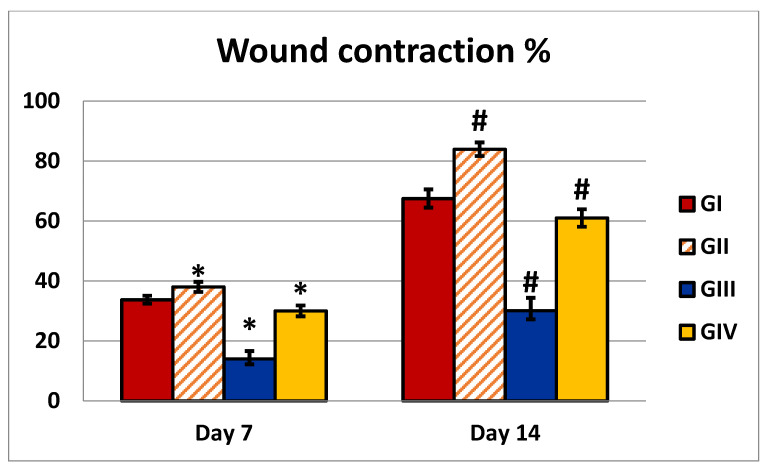
Illustration of an analysis of wounds that were either untreated or treated with DEEL, MRSA, or MRSA + DEEL. Percentage of wound area contraction (wound contraction%) obtained on days 7 and 14. GI: wounded rats treated with normal saline; GII: wounded rats treated with DEEL; GIII: wounded rats infected with MRSA; GIV: wounded rats infected with MRSA and treated with DEEL of *C. macrocarpa*. Symbols * and # indicate that the group is significant in comparison to the control group (GI) at *p* ≤ 0.001 after 7 and 14 days, respectively.

The in vitro antibacterial activity of DEEL was the most potent in comparison with MEDL and TMEL; thus, the antibacterial activity of DEEL was tested in vivo. When DEEL was used to treat wounds, the percentage of wound contraction was significantly higher than in the control group. In addition, when compared to the MRSA-infected group, the infected group treated with DEEL showed a significant increase in wound contraction (Table 3). A significant reduction in the size of the injured area indicated rapid recovery. Figure 6 demonstrates the percentage of the wound area contraction (wound contraction %), collected on days 7 and 14.

#### 2.4.2. Histological Results (H&E)

**Figure 7:**
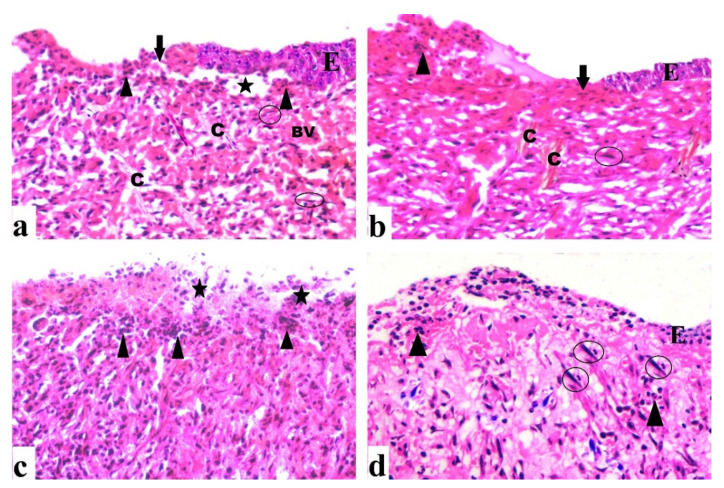
Sections were taken from a skin wound on day 7. (**a**) An untreated wound demonstrated epidermal loss (arrow), as well as dermal and epidermal separation (star). Inflammatory cells (arrowhead) are apparent, as are several fibroblast cells (circle), as well as congested blood vessels (BV) and regular collagen fibers (C). (**b**) A wound treated with DEEL showed epidermal loss (arrow), epidermal layer regeneration (E) with inflammatory cells (arrowhead), collagen fibers (C), and several fibroblast cells (circle). (**c**) An MRSA-infected wound displayed extensive inflammatory cells (arrowhead) as well as necrotic tissue (star). (**d**) MRSA-infected wound + DEEL treatment showed wound re-epithelialization (E) with significant inflammatory cells (arrowhead), aggregation, and fibroblast proliferation (circle) (H&E X200).

**Figure 8:**
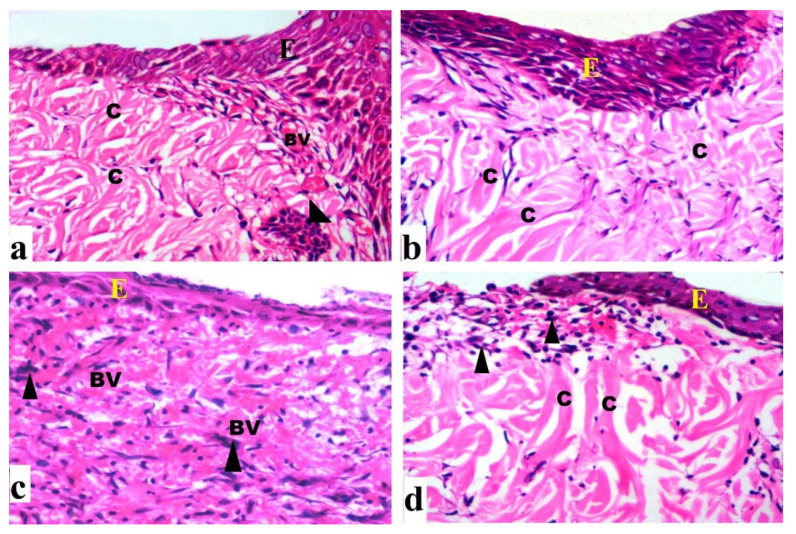
Sections were taken from a skin wound on day 14 (**a**) An untreated wound indicating aggregation of inflammatory cells (arrowhead), collagen fibers (C) that are regular, partial re-epithelialization (E), and blood capillaries (BV). (**b**) DEEL-treated wound with newly formed capillaries, increased fibroblast proliferation, dense regular collagen fibers, and re-epithelialization. (**c**) An MRSA-infected wound displaying poor granulation, numerous inflammatory cells, few fibers, and little re-epithelialization. (**d**) The wound infected with MRSA and treated with DEEL had an incomplete thin regenerated epidermis and collagen fibers (H&E X200).

By day 7, the control group revealed extensive epidermal loss with dermal and epidermal separation occurring on the wound surface. There were inflammatory cells with few fibroblast cells, congested blood vessels, and minimal epidermal growth (Figure 7a). There was epidermal layer regeneration with the less cellular inflammatory response after 14 days, formation of granulation tissue with many cells and some fibers, and moderate collagen deposition (Figure 8a). The group treated with DEEL showed reduced cellular infiltration and regeneration of thin epidermis. Some immature collagen fibers were seen in the dermis after 7 days (Figure 7b). Rats exhibited an incomplete epidermal layer after 14 days. Also, the granulation tissue had newly generated capillaries and higher levels of fibroblast proliferation (Figure 8b). MRSA-treated wound sections after 7 days showed enhanced inflammatory cellular infiltration with necrotic tissue (Figure 7c). By day 14, the granulation tissue was poorly structured, with abundant inflammatory cells, few fibers, and minimal re-epithelialization (Figure 8c).

After 7 days of DEEL treatment for MRSA-infected wounds, incomplete re-epithelization with decreased inflammatory cell aggregation was discovered (Figure 7d). There was an incomplete thin regenerated epidermis after 14 days (Figure 8d).

#### 2.4.3. Mallory Trichrome Stain

**Figure 9:**
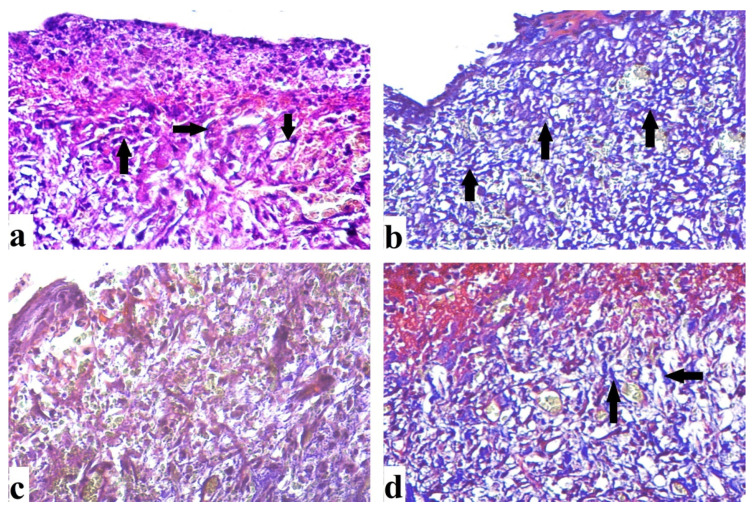
Sections were taken from a skin wound on day 7. (**a**) Untreated wound exhibited thin collagen fibers (arrow) aligned in different orientations. (**b**) DEEL-treated wound showed moderate collagen fibers (arrow). (**c**) An MRSA-infected lesion revealed immature irregular collagen fibers. (**d**) MRSA-infected lesion with DEEL treatment showed moderate irregular collagen fibers (arrow) (Mallory trichromeX200).

**Figure 10:**
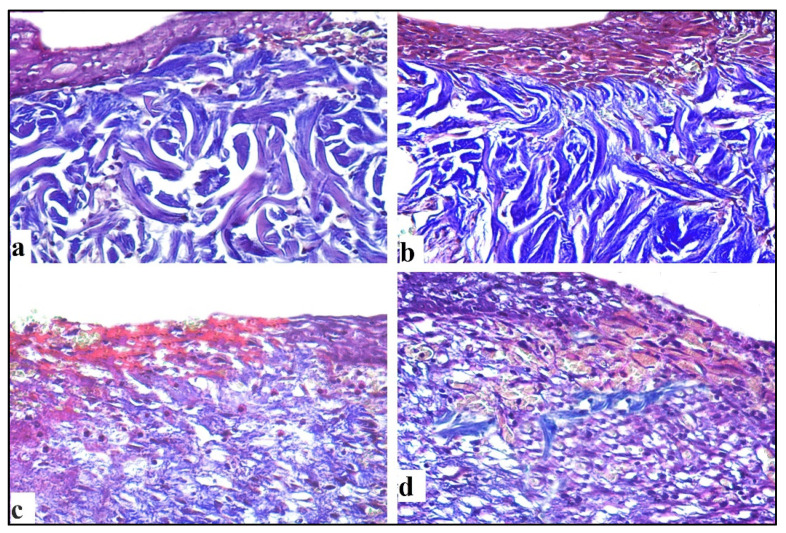
Sections obtained from skin wound on day 14. (**a**) Untreated wound revealed regular moderate collagen fibers. (**b**) DEEL-treated wound exhibited thick regular collagen fibers. (**c**) MRSA-infected wound showed scattering irregular collagen fibers (**d**) MRSA-infected + DEEL treated wound showed immature irregular collagen fibers (Mallory trichromeX200).

**Figure 11:**
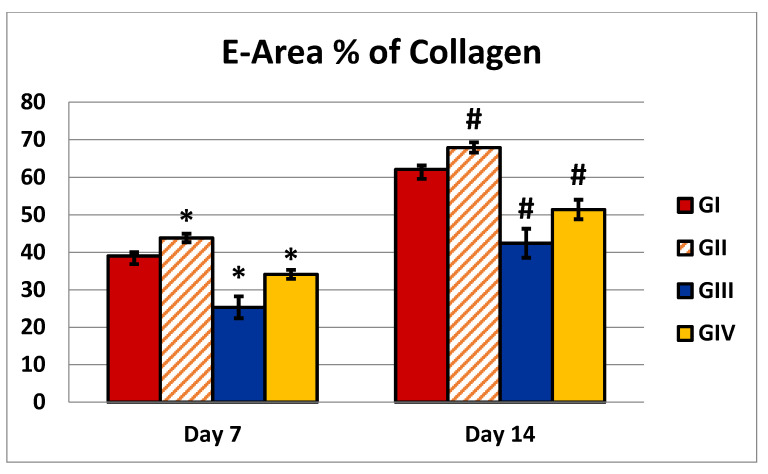
Illustration of an analysis of area % of collagen that was either untreated or treated with DEEL, MRSA, or MRSA + DEEL. Percentage of collagen (area %) obtained on days 7 and 14. GI: wounded rats treated with normal saline; GII: wounded rats treated with DEEL; GIII: wounded rats infected with MRSA; GIV: wounded rats infected with MRSA and treated with DEEL. Symbols * and # indicate that the group is significant in comparison to the control group (GI) at *p* ≤ 0.001 after 7 and 14 days, respectively.

Masson’s trichrome sections of the 7-day control group revealed several distributed collagen fibers aligned in various directions (Figure 9a), while the 14-day wound showed moderate collagen fibers accumulating parallel to the epidermis (Figure 10a). In the *C. macrocarpa* DEEL-treated group, collagen fibers were seen dispersed in numerous orientations (Figure 9b). When compared to the untreated group, there was a considerable increase in thick collagen fibers parallel to the epidermis after 14 days (Figure 10b). In the MRSA-infected group, tiny immature collagen strands were seen in the dermis (Figure 9c). There were scattering irregular collagen fibers after 14 days (Figure 10c). Collagen fibers were found to be thin and uneven after 7 days (Figure 9d) and moderately parallel to the epidermis after 14 days in the infected group treated with DEEL (Figure 10d), which was significantly higher than in the MRSA group (Table 3, Figure 11).

## 3. Discussion

LC-ESI-MS/MS is considered a powerful tool to analyze different plant extracts to reveal their phytochemical content based on their molecular mass and mass fragmentation pattern. The analysis of methanol extract of defatted leaves of *C. macrocarpa* exhibited a tentative identification of 49 compounds of flavones, flavonols, 3′, 4′, 5′- trihydroxy flavonols, methoxylated flavonols, flavanones, and their glycosides. Other compounds recognized include: isoflavone e.g., daidzein-8-*C*-glucoside; biflavonoid e.g., cupressuflavone; labdane diterpenoids e.g., isocupressic acid, acetyl isocupressic acid, and agathadiol; phenolic compounds e.g., shikimic, maleic, quinic acids as well as syringaldehyde, catechin, neohesperidin dihydrochalcone, okanin-4′-*O*-glucoside, rosmarinic acid, phlorizin; and stilbenes e.g., E-3,4,5′-trihydroxy-3′-glucopyranosyl-stilbene.

The ^1^H-NMR spectrum of the first compound displayed the characteristic signals of a flavanone structure represented by signals at δ_H_: 2.55 (1H, dd, *J* = 16.0, 4.0 Hz), 3.78 (1H, dd, *J* = 16.0, 12.0 Hz) for C-3 protons and δH 5.52 (1H, dd, *J* = 12.0, 3.5 Hz) for H-2. Signals for meta-coupled protons at δ 6.12 and 6.14 (*J* = 2.5 Hz) are ascribed to H-6 and H-8. An ABX system was displayed by signals resonating at δ 6.96 (d, *J* = 2.0 Hz) for H-2′, 6.91 (d, *J* = 8.5 Hz) for H-5″, and 6.94 (dd, *J* = 2.0, 8.5 Hz) for H-6″. A methoxy group at C-4′ was compatible with a single signal at δ 3.80 integrated for three protons. Sugar protons ranged from 3.20 to 3.60 ppm (m), while signals for two anomeric protons resonating at δ 4.97 (d, *J* = 7.5 Hz) and 4.69 (d, *J* = 3.0 Hz) suggested the presence of *β*-D-glucose and *α*-L-rhamnose. The ^1^H-NMR established the presence of two hydroxyls, resonating at δ 9.12, 12.01 for OH at C-3ʹ and C-5, respectively.

The ^13^C-NMR spectra revealed the presence of flavanone glycoside by signals resonating at δ_C_ 78.45 and 40.89, which could be ascribed to C- 2 and C-3, respectively. Glucose carbons resonated at δ 99.91, 74.5, 72.53, 70.13, 76.68, and 65.95, while rhamnose carbons (1‴ to 5‴) resonated at δ 101.07, 68.84, 70.68, 76.04, and 71.28, as well as C-6′’’ at δ 18.28. The ESI/MS presented ions at *m/z* 609.144 [M−H]^−^ for C_28_H_34_ O_15_, which is in agreement with the determined structure as hesperidin [25,26]. This is the first report for isolation of hesperidin from *C. macrocarpa* leaves.

The ^1^H-NMR spectrum of the second compound established the characteristic signals for the flavanone structure as represented by signals at δ_H_ 2.88 (1H, dd, *J* = 17.0, 3.5 Hz) and 3.09 ppm (1H, dd, *J* = 17.0, 12.5 Hz) for the two geminal C-3 protons beside the signal at δ 5.21 (1H, dd, J = 12.5, 3.5) for H-2. Signals for meta-coupled protons at δ 6.09 and 6.11 (*J* = 2.5 Hz) were consistent with H-6 and H-8 assignment, respectively. Signals at δ 6.92 (d, *J* = 1.5 Hz) for H-2′, 6.89 (dd, *J* = 1.5, 8.5 Hz) for H-6′, and 6.60 (d, *J* = 8.5 Hz) for H-5′ displayed an ABX system. Multiple signals for sugar protons resonated from δ 3.35 to 4.25. Signals resonating at δ 4.88 (d, *J* = 3.0 Hz) and 4.94 (d, *J* = 7.5 Hz) suggested the presence of α-L-rhamnose and β-D-glucose. The ^13^C-NMR data revealed the presence of flavanone glycoside. Signals resonating at δ_C_ 80.9 and 44.9 could be ascribed to C-2 and C-3, respectively. Glucose carbons resonated at δ 101.12, 74.90, 73.09, 71.70, 77.11, and 66.80, while rhamnose carbons resonated at δ 102.11, 70.32, 72.08, 76.81, 72.40, and 18.01. The ESI/MS of the compound showed ions at *m/z* 595.286 [M−H]^−^ for C_27_H_32_O_15,_ which is in concordance with the determined structure. All spectral data of this compound are consistent with those reported for eriodictioside or eriodictyol- 7-*O*-rutinoside (eriocitrin) [27,28]. This is the first time reporting the isolation of eriodictioside from *C. macrocarpa* leaves.

The global spread of multi-drug resistance among pathogenic bacteria has stimulated enormous interest in research for novel antimicrobial agents from plants [29]. In the current study, we observed that the diethyl ether extract of *C. macrocarpa* leaves showed higher antibacterial activity against MRSA isolates than the methanol extract of defatted leaves and total methanol extract.

The diethyl ether extract was previously investigated phytochemically by GC/MS analysis, which unveiled its phytochemical profile [10]. The major active constituents found in this fraction were abietane diterpenes derivatives, e.g., ferruginol, 13-methyl-13-vinyl-podocarp-7-en-3-one- and 13-isopropyl-podocarpa-6, and 13-diene, as well as monoterpenes which constitute the essential oil part of the diethyl ether extract, e.g., γ-terpinene, α-phellandrene, α-terpinene, camphor, limonene, trans-ocimene, camphene, citronellol, and citronellyl butyrate. [10] Abietane diterpenoids have exerted valuable bioactivity against bacteria and fungias; they were reported to be effective against *Mycobacterium tuberculosis* and *Staphylococcus aureus*, including methicillin-resistant (MRSA) strains and biofilm infection of *S. aureus* [30]. Ferruginol is an aromatic abietane and was reported to exert significant antibacterial effects against *Bacillus subtilis, Staphylococcus aureus*, and *Streptococcus durans* [31]. The active constituents in the essential oil of leaves and branchlets exhibited antimicrobial effects e.g., γ-terpinene [32], α-phellandrene [33], α-terpinene [34], camphor, and limonene [35].

The bacterial growth curve has been commonly used to evaluate the effect of antibacterial agents over a given time (usually 24 hrs) [36]. A significant reduction in the growth of 48.78% of the tested isolates was observed after treatment with DEEL. This may indicate that the antibacterial activity of this extract was associated with a variety of physiological factors in the bacterial cell [37], which needs further studies to explore the potential impact of the extract on different cellular events.

Efflux pumps are proteins located in the bacterial cell membrane. Their function is to transfer toxins out of the cell; thus, they confer cellular protection from many toxins. Efflux pumps are considered the main mechanism of resistance to many antibiotics [38]. We observed that 29.26% of MRSA isolates exhibited a decrease in efflux pump activity after treatment with DEEL. *S. aureus* expresses many efflux pumps that help confer antibiotic resistance. The most common efflux pumps of *S. aureus* are encoded by *nor*A, *nor*B, and *nor*C [39]. For further explanation of the impact of DEEL on the efflux activity of the twelve MRSA isolates that exhibited a decrease in the efflux pump activity by the EtBr cartwheel method, qRT-PCR was used. We observed that 66.66% (8 out of 12 isolates) and 50% (6 out of 12 isolates) showed a significant down expression of *nor*A and *nor*B genes, respectively, after treatment. A nonsignificant change in the expression level of *the nor*C gene was observed after treatment. Many researchers have focused on the detection of efflux pump inhibitors from plant sources [38,40,41,42]. Such efflux inhibitors from a natural source can be used to decrease antibiotic resistance among pathogenic bacteria [38].

The bacterial cell membrane is a major target for many new antibacterial compounds and dissipation of the bacterial cell membrane potential may be the sole mechanism of action, or it can contribute to the potency of the studied antibacterial compound [43]. Thus, we examined the membrane depolarization in MRSA isolates before and after treatment with DEEL. A nonsignificant change in membrane depolarization after treatment was observed.

Scanning electron microscopy is commonly used in microbiological research to study the ultrastructural changes that occur in the morphology of bacterial cells after treatment with antimicrobial agents [44]. Therefore, SEM was utilized in the current study as it can produce images for the external cell morphology and surface characteristics with higher resolution compared to the light microscope. We observed that the bacterial cells after treatment with DEEL shrunk with degradation to the cell walls.

Wound healing is a vital biological process that happens when a variety of cell types and their products interact synergistically [45]. Bacterial infection, however, can inhibit and impede natural wound healing. *S. aureus* is a dangerous bacterium that can cause wound infection [46]. From the in vitro antibacterial study, it was found that diethyl ether of *C. macrocarpa* leaves exhibited the best activity; as a result, we investigated its efficacy on a rat model with an MRSA-infected cutaneous wound.

According to a histological examination, 7 days of untreated wounds showed cellular infiltration with thin collagen fibers. After 14 days, there was a reduction in inflammatory cells, thick collagen deposition, and partial re-epithelialization. Our findings were consistent with those of reported data [47].

Our in vivo study results demonstrated that treatment of DEEL of *C. macrocarpa* was highly significant against cutaneous wound infection in a rat model. It significantly improved wound contracture and healing. Furthermore, it appeared to improve healing by regenerating the epidermis, maturing granulation tissue, and reducing inflammatory cell infiltration associated with collagen fibers deposition when compared to with the MRSA-infected group. These findings were consistent with those that showed that *C. macrocarpa* leaves inhibited the production of proinflammatory cytokines such as PGE2, TNF-a, IL-1b, and IL-6, hence preventing chronic inflammation and enhancing wound healing [9]. The histopathology finding was also consistent with previous findings by Saad et al. in 2017 [5].

MRSA-infected wounds were shown to have an abundance of inflammatory cells, few collagen fibers, little re-epithelialization, and inadequate granulation tissue, as well as a significant reduction in wound contracture as compared with untreated wounds. According to researchers, *S. aureus* infection causes cutaneous collagen breakdown, which leads to impaired granulation tissue and, as a result, delayed wound healing [48].

The cell viability SRB assay of DEEL against HSF normal cell line was performed. IC_50_ of DEEL was 21.3 ± 3.41 µg/mL in comparison with doxorubicin (IC_50_ = 4.36 ± 0.52 µg/mL) as a positive control.

Finally, our results provided light on the reusing of *C. macrocarpa* as an efficient and novel topical therapy to treat MRSA. The current study laid the groundwork for future clinical trials as an antibacterial drug to treat skin infections caused by pathogenic MRSA isolates.

## 4. Materials and Methods

### 4.1. Plant Material

*Cupressus macrocarpa* leaves with branchlets were collected and dried at room temperature in May 2017 from a nursery at Shebin El-Kom, El-Menoufia Governorate. The plant was recognized by Prof. Mohammed Ibrahim Fotoh, Professor of Ornamental Horticulture and Landscape Design. A voucher sample (PG00411) was deposited at Herbarium of the Department of Pharmacognosy, Faculty of Pharmacy, Tanta University. The different extracts were carried out by the cold maceration method. The dried leaves with branchlets (2 kg) of *C. macrocarpa* were extracted first with 4 L of petroleum ether for 3 days, three times (4 L × 3 times), and then with 5 L of 95% methanol three times (5 L × 3 times) for 3 days each. Both extracts were evaporated under reduced pressure. The yield was 7.5% and 6.2% for petroleum ether and methanol extracts, respectively.

### 4.2. Method of Isolation of Flavonoids

The dried methanol extract was suspended in a mixture of methanol and water (2:1). The formed suspension was successively extracted with dichloromethane, ethyl acetate, and *n*-butanol. Extracts of methylene chloride, ethyl acetate, and *n*-butanol were completely evaporated under a vacuum at 40 °C to obtain a dry residue of each solvent. Ethyl acetate (4 g) was applied to a silica gel column chromatography (160 g, ϕ 5 × 30 cm), and fractions of 50 mL volume were collected. Elution was undertaken with a successive gradient of CH_2_Cl_2_–MeOH mixtures of increasing polarities. The CH_2_Cl_2_–MeOH (85:15) eluates, A21 (325 mg) and A23 (245 mg), were chromatographed to column chromatography using silica gel (20 g, ϕ 2 × 15 cm). The collected fractions were 15 mL each, which were eluted successively with an increasing gradient of the polarity of CH_2_Cl_2_/MeOH mixture. Subfraction B19 eluted from A21 sub-column at (90:10) of CH_2_Cl_2_: methanol was purified on Sephadex LH-20 (15 g, ϕ, 2 × 12 cm) with MeOH to afford the first compound (10 mg). Subfraction C15 eluted from A23 sub-column at (88:12) of CH_2_Cl_2_: methanol was applied to isocratic CC (10 g, ϕ, 1.2 × 10 cm) on silica gel with CHCl_3_: MeOH (95:5) to give a yellowish residue; this residue was subjected to Sephadex LH-20 (15 g, ϕ, 2 × 10 cm) with MeOH to give the second compound (8 mg). Structure determination was undertaken by A JEOL ECA-500 II NMR spectrometer, which was used to record NMR spectra. NMR samples were analyzed at 500 MHz for ^1^H and 125 MHz for ^13^C. Samples were dissolved in DMSO-d_6_.

### 4.3. LC-ESI-MS/MS Analysis of C. Macrocarpa Leaves Extract

#### 4.3.1. Preparation of Plant Sample

A total of 100 g of dried leaves powder was defatted by maceration in petroleum ether at room temperature. After complete exhaustion, methanol was added to the powder, and after extraction, methanol was evaporated under a vacuum at 40 °C. A weighed portion of the residue (50 mg) was reconstituted in a 1 mL solution of deionized water, methanol, and acetonitrile (50: 25: 25). The dissolved sample was vortexed for 2 min, ultra-sonicated for 10 min, and centrifuged for another 10 min at 1000 rpm. Dilution was carried out with the reconstitution solvent to inject 10 µL of the sample solution at a concentration of 1 µg/µL.

#### 4.3.2. LC-ESI-MS/MS Method

Compounds in the crude extracts were identified by Proteomics and Metabolomics Unit, Children’s Cancer Hospital (57357), Basic Research Department, Cairo, Egypt. The crude extracts were reconstituted in DI-Water, methanol, and acetonitrile (50:25:25) and analyzed by LC-ESI-MS/MS using ExionLC^TM^ AD UPLC, coupled with TripleTOF 5600+ Time-of-Flight Tandem Mass Spectrometer (AB SCIEX). The injection concentration was 1 μg/mL and volume 10 μL. The column used was the XSelect HSS T3 XP column (Waters, 2.1 mm × 150 mm) at 40 °C. The flow rate was 300 µL/min of the mixture of 1% methanol in 5 mM ammonium formate buffer (pH 8) (A) and acetonitrile (B). Gradient elution was used as the following: isocratic elution with 90% of solvent A and 10% of acetonitrile for 1 min, gradient elution with 90 to 10% solvent A and 10% to 90% of acetonitrile for 20 min, 90% of acetonitrile for 4 min, and then returned to the initial condition (10% of acetonitrile) for 3 min. Mass spectra were acquired under negative-ion mode in an information-dependent mode with an automatic switch between a full scan (50–1000 *m*/*z*) and up to 15 information-dependent MS/MS scans. The intensity threshold to trigger an MS/MS scan was set to 10^4^ ppm. The MS1 and MS2 tolerance values were 0.01 and 0.05 mass units, respectively. The maximum ion monitoring time was 3000 ms. The ion spray voltage was set to 4500 V, the turbo spray temperature 500 °C, nebulizer gas 45 psi, heater gas 45 psi, and declustering potential 80 V, and normalized collision energy 35 V. The dynamic exclusion was applied using a setting of 10 seconds. Raw data files were loaded into MS-DIAL 3.52 (http://prime.psc.riken.jp/) [49] for data-independent MS/MS deconvolution. Compounds were identified with > 70% probability using a MS1 and MS2 tolerance of 0.2 mass unit to be accepted as positive identifications. PeakView was used for feature or peaks extraction from total ion chromatogram (TIC). Based on that, the features should have a signal-to-noise ratio greater than 5 (non-targeted analysis) as well as features intensities of the sample-to-blank ratio as greater than 3.

### 4.4. Materials and Methods of Antibacterial Study of C. Macrocarpa Leaves Extracts

#### 4.4.1. Bacterial Isolates and Chemicals

A total of 41 MRSA isolates were collected from clinical samples from different departments of Tanta University Hospital. The clinical samples were originally taken from patients for proper diagnosis and treatment, thus ethical approval was not required. The bacterial isolates were viewed microscopically and were identified by standard biochemical tests (MacFaddin, 1976). MRSA isolates were detected phenotypically, using the cefoxitin disk diffusion (30 μg) method [50] and genotypically, using PCR for detection of the *mec*A gene [51]. *Staphylococcus aureus* (ATCC 29231) was used as a reference strain.

#### 4.4.2. In vitro Antibacterial Activity Testing

Three different portions of 100 g of leaves and branchlets dried powder, were macerated at room temperature. The first portion was macerated in diethyl ethyl ether (DEEL), the second portion in light petroleum ether tell exhaustion (defatted leaves) then methanol (MEDL), and the third portion in methanol (TMEL) only. Each extract was evaporated under a vacuum to obtain three different residues. The in vitro antibacterial activity of the obtained residues was carried out by agar well diffusion method [52]. Approximately 100 µL of each bacterial suspension was distributed on the surface of plates containing Muller-Hilton agar. Then, five wells were punched off using a cork-borer and each well was filled with 100 μL (1024 µg/mL) of each of the following: DEEL, MEDL, and TMEL (reconstituted in DMSO). The plates were incubated at 37 °C for 24 h using DMSO as a negative control and vancomycin (30 μg/mL) as a positive control.

##### Determination of MICs

The MIC values were determined for DEEL, MEDL, and TMEL by broth microdilution method using DMSO for dilutions [50]. A well containing a bacterial suspension without the extract (a positive control) and another well containing broth without any bacteria (a negative control) were incorporated in each microtitration plate. The MIC values of the extracts were identified for each bacterial isolate as the lowest extract concentration, which showed complete inhibition of bacterial growth (i.e., absence of turbidity). The subsequent tests (in vitro and in vivo) were accomplished before and after treatment of MRSA isolates with sub-inhibitory concentrations (i.e., 0.5 MIC values ranged from 1 to 4 µg/mL) of DEEL (the most potent).

##### Growth Curve Assay

This assay was conducted to assess the impact of DEEL on the growth of MRSA (at 0.5 MIC values ranging from 1 to 4 µg/mL) [53]. The optical density (OD) of MRSA (before and after treatment) was measured at 620 nm using 1800 UV–Vis spectrophotometer (SHIMADZU, Kyoto, Japan) at time intervals of 0, 2, 4, 6, 8, and 24 h. The growth curves were constructed by plotting log OD_620_ versus the sampling time (h).

##### Efflux Assay

This assay was conducted before and after treatment with DEEL (at 0.5 MIC values ranging from 1 to 4 µg/mL), using the EtBr cartwheel method [39,54], and the reference strain was used as a negative control. Each bacterial suspension was swabbed as redial lines onto tryptic soy agar (TSA) plates supplied with concentrations of EtBr ranging from 0.5 to 2.5 mg/L, and they were incubated for 16 hrs at 37 °C. The minimum concentration of EtBr resulted in the production of fluorescence by the bacterial isolates and was recorded using a UV-Vis transilluminator (SHIMADZU, Kyoto, Japan). MRSA isolates were classified based on the detected minimum concentration of EtBr. Isolates emitting fluorescence at 0.5 mg/L EtBr were considered to have negative efflux activity; isolates emitting fluorescence at 1–2.0 mg/L EtBr were considered to have intermediate efflux activity, and isolates emitting fluorescence at 2.5 mg/L EtBr were considered to have positive efflux activity.

##### QRT-PCR

The expression levels of the efflux pump genes (*nor*A, *nor*B, and *nor*C) [55] were detected after treatment with DEEL using qRT-PCR. Briefly, after centrifugation of the overnight cultures of MRSA isolates (with and without treatment), the pellets were immediately utilized for total RNA extraction using the Purelink^®^ RNA Mini Kit (Thermo SCIENTIFIC, Waltham, USA) as described by the manufacturer. The purified RNA was rapidly retrotranscribed into cDNA using a power cDNA synthesis kit (iNtRON Biotechnology, Korea) as recommended by the manufacturer. qRT-PCR was accomplished using the Rotor-Gene Q 5plex machine (Qiagen, Hilden, Germany). The qRT-PCR was conducted to calculate the fold changes in the expression of the tested genes using the housekeeping gene 16S rRNA as an endogenous control [51]. The used primers are listed in Table S1. The relative levels of target gene expression were quantified using the 2^−ΔΔCt^ method (the expression levels of isolates before treatment were set to be 1) [56]. Only genes with ≥two-fold changes (either increased or decreased) were regarded to be statistically significant [57].

##### Membrane Depolarization Assay

This assay was performed after staining the bacterial cells (before and after treatment with DEEL using DiBAC4(3), a fluorescent molecular probe) as previously described [58]. The utilized instrument to analyze the cellular staining was the FACSVerse flow cytometer (BD Biosciences, Franklin Lakes, NJ, USA).

##### Examination of Morphology by SEM

The cell morphology of MRSA isolates was examined by SEM (Hitachi, Chiyoda, Japan) before and after treatment with DEEL as described by McDowell and Trump [59].

#### 4.4.3. In Vivo Antibacterial Activity Testing

In this experiment, forty adult male albino rats weighing between 180 and 220 g were used. Animals were obtained from the animal house at Menoufia University’s Faculty of Medicine and were acclimatized under laboratory settings for one week before the experiment began. Rats were housed in a safe standard habitat at room temperature, with an unrestricted meal and water supply. Four groups, each with ten animals, were constructed. After removing the dorsal hair and sterilizing with 10% povidone-iodine, a full-thickness excision wound measuring 1.5 × 1.5 cm was created on the mid-back of each animal.

1. Group I (control group): wounds were treated daily with 20% DMSO in normal saline (0.9% (*w*/*v*) NaCl freshly prepared).
2. Group II (*C. macrocarpa* group): wounds were treated daily with a thin layer of DEEL in 20% DMSO in normal saline (1 mg/mL, 1 mm thickness).
3. Group III (MRSA-infected group): MRSA was used to contaminate the wounds with 10 μL of the bacterial suspension (10^6^ CFU).
4. Group VI (MRSA+ *C. macrocarpa* treated group): wounds were infected with MRSA and treated daily with a thin coating of DEEL in 20% DMSO in normal saline (1 mg/mL, 1 mm thickness).

##### Histological Study

On day 7, five rats from each group were sacrificed, and the remaining animals were slaughtered on day 14. The skin wound tissues were immediately extracted and handled in 10% formal saline for histological examinations. Tissues fixed in paraffin were sliced into 5 μm thick sections and submitted to Haematoxylin and Eosin (H&E) stain and Mallory’s trichrome stain for collagen fibers procedures [60].

##### Morphometric Analysis

Ten different isolated fields from each segment were measured for quantitative evaluation using a Leica DML B2/11888111 microscope equipped with a Leica DFC450 camera (Leica Microsystems, Wetzlar, Germany). Image J software version K1.45 was used to estimate the measured variance. H&E and Mallory’s trichrome were used to collect data.

Wound closure % = (initial wound size−wound at the time of taking the image)/initial wound size) × 100 [61] mean area percentage of collagen fibers with sections stained with Mallory’s Trichrome (×200).

### 4.5. Chemical Reagents and Cell Line

Methanol, formic acid, and sodium hydroxide for PH adjustment (Fisher Scientific, Loughborough, UK); vancomycin, acetonitrile, and ammonium formate (Sigma-Aldrich, Darmstadt, Germany); water (Milli-Q) (Millipore, Burlington, VT, USA). Sigma Aldrich provided sulfoxide (DMSO), doxorubicin, Sulfo-Rhod-amine-B stain (SRB) (3-(4,5-dimethylthiazol-2-yl)-2,5-diphenyltetrazolium bromide), and all the analytical grade solvents used in this study. HSF cell line was obtained from Nawah Scientific Inc. (Mokatam, Cairo, Egypt).

### 4.6. Cytotoxicity Assay

Cell viability was assessed by SRB assay. In 96-well plates, aliquots of 100 μL cell suspension (5 × 10^3^ cells) were incubated in a complete medium for 24 h. Another aliquot of 100 μL media containing the extract at various concentrations was used to treat the cells. After 72 hrs of exposure to the extract, the cells were fixed by replacing the medium with 150 μL of 10% trichloroacetic acid (TCA) and incubating for one hour at 4 °C. After removing the TCA solution, the cells were washed five times with distilled water. Aliquots of 70 μL of SRB solution (0.4 percent *w*/*v*) were added and incubated at room temperature for 10 min in the dark. Plates were washed three times with 1% acetic acid and air-dried overnight. The absorbance was measured at 540 nm using a BMG LABTECH^®^- FLUOstar Omega microplate reader (BMG LABTECH, Ortenberg, Germany) after 150 μL of TRIS (10 mM) was added to dissolve protein-bound SRB stain [24].

### 4.7. Statistical Analysis

All the conducted tests were carried out in triplicate. Data are presented as means ± standard deviation (SD) using SPSS software version 26 (IBM Corp., New York, NY, USA). The varied parameters acquired from separate groups were compared using *t*-test (ANOVA) and Bonferroni’s post hoc test to establish statistical significance. *p* < 0.001 was used to determine the statistical significance of the data.

## 5. Conclusions

The elucidation of 49 different compounds was carried out tentatively for the defatted methanol extract of *C. macrocarpa* leaves using LC-ESI-MS/MS. Isolation of two flavanones, hesperidin and eriocitrin, was conducted for the first time. DEEL demonstrated the best antibacterial activity against 48.78% of MRSA clinical isolates. It significantly reduced the efflux activity by 29.26% as well as downregulating the expression of *nor*A and *nor*B genes in 66.66% and 50% of the clinical isolates, respectively, and significantly affected the bacterial cell morphology. The in vivo study results exhibited that topical treatment with DEEL is highly efficient against cutaneous wound infections in a rat model., which significantly improved wound healing through regenerating the epidermis, maturing granulation tissue, and reducing inflammatory cell infiltration, in comparison with the MRSA-infected group. The in vivo study on rats showed no adverse reactions in all rates during the 14 days. Generally, from the obtained results, we can conclude that DEEL may provide a promising source to develop a curative antibacterial drug against the problematic MRSA bacteria.

## Figures and Tables

**Table 1:** Phytochemical profiling of *C. macrocarpa* leaves extract by LC-ESI-MS/MS in negative mode.

No.	Rt (min.)	[M − H]^−^ *m/z*	MS^2^ *m/z*	Formula	Identification
1	1.17	115.000	115.081, 71.017	C_4_H_4_O_4_	Maleic acid
2	1.19	625.063	625.071, 429.022, 369.071, 346.077, 301.012	C_27_H_30_O_17_	Quercetin-3,4′-*O*-β-diglucopyranoside
3	1.21	173.046	173.044, 155.060, 140.924, 118.587, 109.024, 93.033	C_7_H_10_O_5_	(-)-Shikimic acid
4	1.22	191.056	191.054, 173.046, 133.013, 111.044, 109.024, 93.032	C_7_H_12_O_6_	D-(-)-Quinic acid
5	1.23	317.053	317.055, 281.093, 279.106, 249.00, 191.056	C_15_H_10_O_8_	Myricetin
6	1.59	163.038	163.039, 119.049, 91.014	C_9_H_8_O_3_	3-(4-hydroxyphenyl)prop-2-enoic acid (p-coumaric acid)
7	4.10	577.172	577.156, 532.902, 269.104	C_27_H_30_O_14_	Rhoifolin
8	3.45	447.188	447.180, 401.180, 301.151, 239.127, 179.070, 151.046, 119.035	C_21_H_20_O_11_	Quercitrin
9	4.60	417.139	417.134, 371.169, 285.92, 209.0788	C_20_H_18_O_10_	Kaempferol-3-*O*-alpha-L-arabinoside
10	4.66	461.148	461.127, 392.912, 285.103	C_21_H_18_O_12_	Kaempferol-3-*O*-Glucuronide
11	4.80	283.096	283.090, 179.100, 171.098, 146.956, 73.030	C_16_H_12_O_5_	Acacetin
12	4.84	289.070	289.067, 245.084, 205.044, 187.040, 179.046, 151.044, 123.052	C_15_H_14_O_6_	Catechin
13	5.10	183.025	183.034	C_8_H_8_O_5_	3,4-Dihydroxymandelate
14	5.65	431.191	431.192, 383.924, 285.213, 165.095, 159.034, 149.047, 125.023, 119.035	C_21_H_20_O_10_	Kaempferol-3-*O*-alpha-L-rhamnoside
15	5.67	385.172	385.184, 248.947, 223.131, 205.121, 190.970, 179.053, 161.042, 149.052, 119.035, 71.016	C_17_H_22_O_10_	1-*O*-*β*-D-glucopyranosyl sinapate
16	5.89	463.169	463.171, 403.151, 317.103, 301.093, 208.087, 194.033, 150.056	C_21_H_20_O_12_	Myricitrin
17	6.01	449.108	449.102, 403.165, 381.167, 287.03293, 269.042, 179.002, 151.00, 107.022	C_21_H_22_O_11_	Eriodictyol-7-*O*-glucoside
18	6.260	609.143	609.146, 563.234, 496.863, 285.201, 315.043 300.03	C_27_H_30_O_16_	Luteolin-3′, 7-di-*O*-glucoside
19	6.29	433.111	433.115, 271.053, 151.036, 119.043	C_21_H_22_O_10_	Naringenin-7-*O*-glucoside
20	6.69	435.092	435.084, 417.083, 389.214, 309.053, 285.040, 178.993, 151.042, 125.023	C_21_H_24_O_10_	Phlorizin
21	6.79	449.102	449.114, 431.080, 342.910, 303.049, 285.045, 276.910, 151.00	C_21_H_22_O_11_	Okanin-4′-*O*-glucoside
22	6.97	595.169	595.286, 548.851, 480.844, 315.058, 287.030	C_27_H_32_O_15_	Eriodictyol-7-*O*-rutinoside
23	6.99	445.171	445.169, 269.142, 112.989	C_21_H_18_O_11_	Baicalein-7-*O-*glucuronide
24	6.98	623.063	623.208, 579.156, 532.918, 315.036	C_28_H_32_O_16_	Isorhamnetin-3-*O-*rutinoside
25	7.15	609.521	609.144, 301.027	C_28_H_34_O_15_	Hesperidin
26	7.24	433.076	433.077, 389.175, 385.201, 326.928, 301.037, 300.030, 287.049, 271.025	C_20_H_18_O_11_	Quercetin-3-*O*-Arabinoside
27	7.48	477.099	477.098, 431.222, 364.927, 331.042, 315.041, 300.043	C_22_H_22_O_12_	Isorhamnetin-3-*O*-glucoside
28	7.52	507.111	507.102, 489.186, 461.112, 345.057, 326.930, 315.061, 286.951, 269.121	C_23_H_24_O_13_	Syringetin-3-*O*-glucoside
29	7.53	447.092	447.088, 401.248, 301.034, 300.020, 285.040, 271.026	C_21_H_20_O_11_	Quercetin-7-*O*-rhamnoside
30	7.57	433.079	433.080, 349.103, 326.928, 301.036, 300.023, 269.047, 178.993, 152.010	C_20_H_18_O_11_	Quercetin-3-D-xyloside
31	7.67	405.061	405.093, 369.248, 231.0, 209.066, 191.056, 137.020	C_20_H_22_O_9_	E-3,4,5′-Trihydroxy-3′-glucopyranosyl-stilbene
32	7.70	463.088	463.119, 394.907, 354.924, 331.054, 316.021, 301.066, 286.934	C_21_H_20_O_12_	Quercetin-4′-glucoside
33	7.87	431.097	431.092, 385.175, 341.181, 299.023, 269.035	C_21_H_20_O_10_	Apigenin-7-*O*-glucoside
34	7.90	447.182	447.096, 401.202, 285.040, 112.989	C_21_H_20_O_11_	Luteolin-7-*O*-glucoside
35	7.82	415.197	415.195, 369.212, 253.165, 179.063, 161.042, 113.020	C_21_H_20_O_9_	Daidzein-8-*C*-glucoside
36	8.22	507.111	507.112, 462.907, 445.205, 430.890, 394.919, 371.207, 345.124, 329.041, 286.932	C_23_H_24_O_13_	Syringetin-3-*O*-galactoside
37	8.39	301.123	301.118	C_16_H_14_O_6_	Hesperetin
38	9.57	537.083	537.021, 518.951, 493.504, 468.105, 255.025, 248.956, 213.018, 197.059	C_30_H_18_O_10_	Cupressuflavone
39	9.76	301.037	301.036, 255.221, 243.039, 178.997, 151.0094	C_15_H_10_O_7_	Quercetin
40	10.08	299.056	299.055, 284.042	C_16_H_12_O_6_	3,5,7-Trihydroxy-4′-methoxy-flavone
41	10.12	359.169	359.176	C_18_H_16_O_8_	Rosmarinic acid
42	10.23	181.047	181.049, 166.031, 138.030, 112.986	C_9_H_10_O_4_	Syringaldehyde
43	10.29	271.060	271.064, 177.022, 151.004, 119.046, 107.020	C_15_H_12_O_5_	Naringenin
44	10.85	611.113	611.117, 565.214, 504.961	C_28_H_36_O_15_	Neohesperidin dihydrochalcone
45	10.96	285.039	285.038, 185.068	C_15_H_10_O_6_	Luteolin
46	11.23	315.109	315.169, 300.028, 297.187, 283.022, 269.246, 246.898, 235.177, 141.018	C_16_H_12_O_7_	3′-Methoxy-4′,5,7-trihydroxyflavonol
47	15.14	319.227	319.226, 318.20, 255.209, 248.572, 164.252	C_20_H_32_O_3_	Isocupressic acid
48	19.60	361.238	361.237, 319.231, 301.218, 283.165	C_22_H_34_O_4_	Acetylisocupressic acid
49	23.33	305.248	305.249, 304.280, 166.095	C_20_H_34_O_2_	Agathadiol

**Table 2:** Efflux activity of MRSA isolates, measured by EtBr cartwheel method, before and after treatment with DEEL.

Efflux Activity	No. of Isolates before Treatment	No. of Isolates after Treatment
Negative efflux activity	8	21
Intermediate efflux activity	14	13
Positive efflux activity	19	7

**Table 3:** Percentage of wound contraction and area percentage of collagen in different groups.

X^−^ ± SD					
	GI	GII	GIII	GIV	*p*-Value
Wound contraction % (7 days)	33.73 ± 1.32	38 ± 1.7	14 ± 2.58	30 ± 1.82	*P*1 = 0.0001 *P*2 = 0.0001 *P*3 = 0.0001 *P*4 = 0.0001
Wound contraction % (14 days)	67.5 ± 3.03	83.9 ± 2.28	30.1 ± 4.28	61 ± 2.9	*P*1 = 0.0001 *P*2 = 0.0001 *P*3 = 0.0001 *P*4 = 0.0001
Area % of collagen (7 days)	39 ± 2.16	43.8 ± 1.13	25.3 ±2.9	34.1±1.19	*P*1 = 0.0001 *P*2 = 0.0001 *P*3 = 0.0001 *P*4 = 0.0001
Area % of collagen (14 days)	62.1 ±2.51	67.9 ±1.37	42.4 ±3.86	51.4 ±2.59	*P*1 = 0.0001 *P*2 = 0.0001 *P*3 = 0.0001 *P*4 = 0.0001

GI: wounded rats treated with normal saline; GII: wounded rats treated with DEEL; GIII: wounded rats infected with MRSA; GIV = wounded rats infected with MRSA and treated with DEEL; X^−^: mean value; SD: standard deviation; significance: (*p*-value ≤ 0.001). ***P*1**: group 2 compared to control; ***P*2**: group 3 compared to control; ***P*3**: group 4 compared to control; ***P*4**: group 4 compared to group 3.

## Data Availability

The data presented in this study are available on request.

## Supplementary Materials

The following are available online at https://www.mdpi.com/article/10.3390/antibiotics10080890/s1, Figure S1: total ion chromatogram (TIC) of methanol extract of *C. macrocarpa* leaves (negative mode). Figure S2 (a,b,c,d): structure of flavonoids and their glycosides identified in *C. macrocarpa* leaves and methanol extract of leaves. Figure S3: compounds tentatively identified of *C. macrocarpa* leaves by LC-ESI-MS/MS (Glu = glucose). Table S1: The sequences of utilized primers.
