# Supplementary material for: Elucidation of Phytochemical Content of Cupressus macrocarpa Leaves: In Vitro and In Vivo Antibacterial Effect against Methicillin-Resistant Staphylococcus aureus Clinical Isolates
Source: Antibiotics (Basel). 2021 Jul 22;10(8):890. doi: 10.3390/antibiotics10080890 (PMC8388636; doi:10.3390/antibiotics10080890)
Supplement: Supplementary file 1 [file antibiotics-10-00890-s001.zip › antibiotics-1282942-supplementary.pdf]

Supplementary data

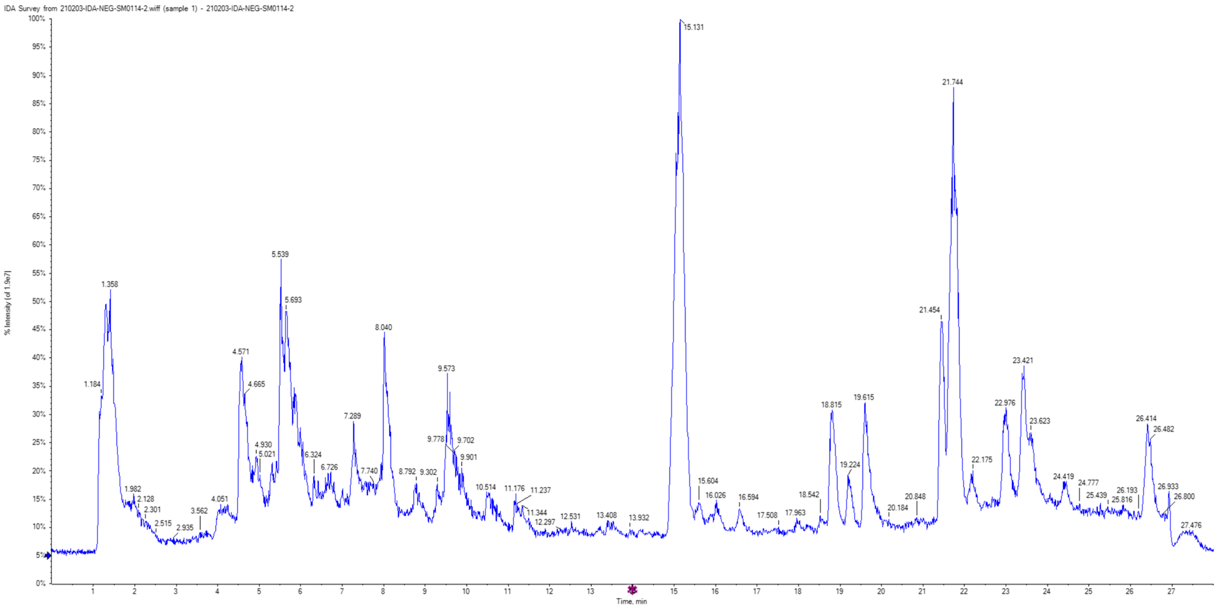

Figure S1. Total ion chromatogram (TIC) of methanol extract of *C. macrocarpa* leaves (negative mode)

Table S1. The sequences of the utilized primers

Gene	Primer	Sequence
*nor*A	**Forward**	5′-GACATTTCACCAAGCCATCAA-3′
	**Reverse**	5′-TGCCATAAATCCACCAATCC-3′
*nor*B	**Forward**	5′-GCTACACCATCAACAGATACAGCAA-3′
	**Reverse**	5′-ACTCAATGCGACGCCAAA-3′
*nor*C	**Forward**	5′-TGGGTTGGAGATGGATTTTC-3′
	**Reverse**	5′-ACAATTAGCCCTGCAACGTC-3′
**16srRNA**	**Forward**	5′-CGTGGAGGGTCATTGGA-3′
	**Reverse**	5′-CGTTTACGGCGTGGACT-3′

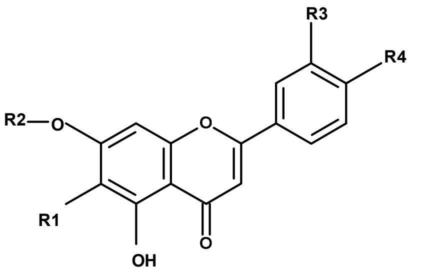

Compound	R1	R2	R3	R4
Luteolin	H	H	OH	OH
Luteolin-7-*O*-glucoside	H	Glu	OH	OH
Luteolin-3',7-diglucoside	H	Glu	O-Glu	OH
Apigenin-7-*O*-neoheperidoside	H	Neo	H	OH
Apegenin-7-*O*-glucoside	H	Glu	H	OH
Acacetin	H	H	H	OCH_3
Baicalein-7-*O*-glucouronide	OH	Gluc	H	H

Figure S2a. Structures of flavones and flavones glycosides identified in *C. macrocarpa* leaves methanol extract of leaves (Glu= glucose, Neo= neoheperidoside, Gluc= glucuronide)

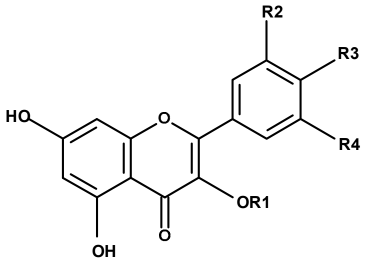

Compound	R1	R2	R3	R4
3, 5, 7-Trihydroxy-4'-methoxyflavone	H	H	OCH_3	H
Myricetin	H	OH	OH	OH
Myricitrin	Rha	OH	OH	OH
Syringetin-3-*O*-glucoside	Glu	OCH_3	OH	OCH_3
Syringetin-3-*O*-galactoside	Gal	OCH_3	OH	OCH_3
Isorhamnetin-3-*O*-glucoside	Glu	OCH_3	OH	H
Isorhamnetin-3-*O*-rutinoside	Rutinoside	OCH_3	OH	H
4',5,7-Trihydroxy-3'-methoxy-flavonol	H	OCH_3	OH	H

Figure S2b. Structures of 3',4',5'-trihydroxy, methoxylated, and glycosidic flavonols identified in *C. macrocarpa* leaves methanol extract (Rha= rhamnose, Glu= glucose, Gal= galactose)

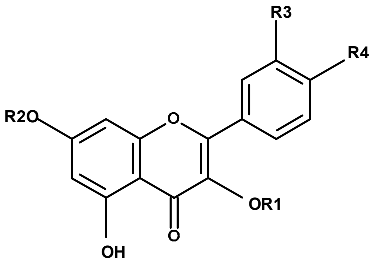

Compound	R1	R2	R3	R4
Quercetin	H	H	OH	OH
Quercitrin	Rha	H	OH	OH
Quercetin-7-*O*-rhamnoside	H	Rha	OH	OH
Quercetin-3-*O*-arabinoside	Ara	H	OH	OH
Quercetin-3,4'-*O*-di-glucoside	Glu	H	OH	O-Glu
Quercetin-3-*O*-xyloside	Xyl	H	OH	OH
Quercetin-4'-*O*-glucoside	H	H	OH	O-Glu
Kampferol-3-*O*-glucuronoid	Gluc	H	H	OH
Kaempferol-3-*O*-rhamnosoide	Rha	H	H	OH
Kaempferol-3-*O*-arabinoside	Ara	H	H	OH

Figure S2c. Structures of flavonols and flavonols glycosides (Rha= rhamnose, Ara= arabinose, Xyl= xylose)

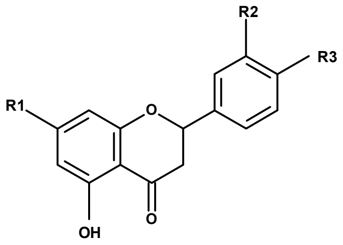

Compound	R1	R2	R3
Hesperetin	OH	OH	OCH_3
Naringenin	OH	H	OH
Naringenin-7-*O*-glucoside	O-Glu	H	OH
Eriodictyol-7-*O*-glucoside	O-Glu	OH	OH

Figure S2d. Structures of flavanones and their glycosides

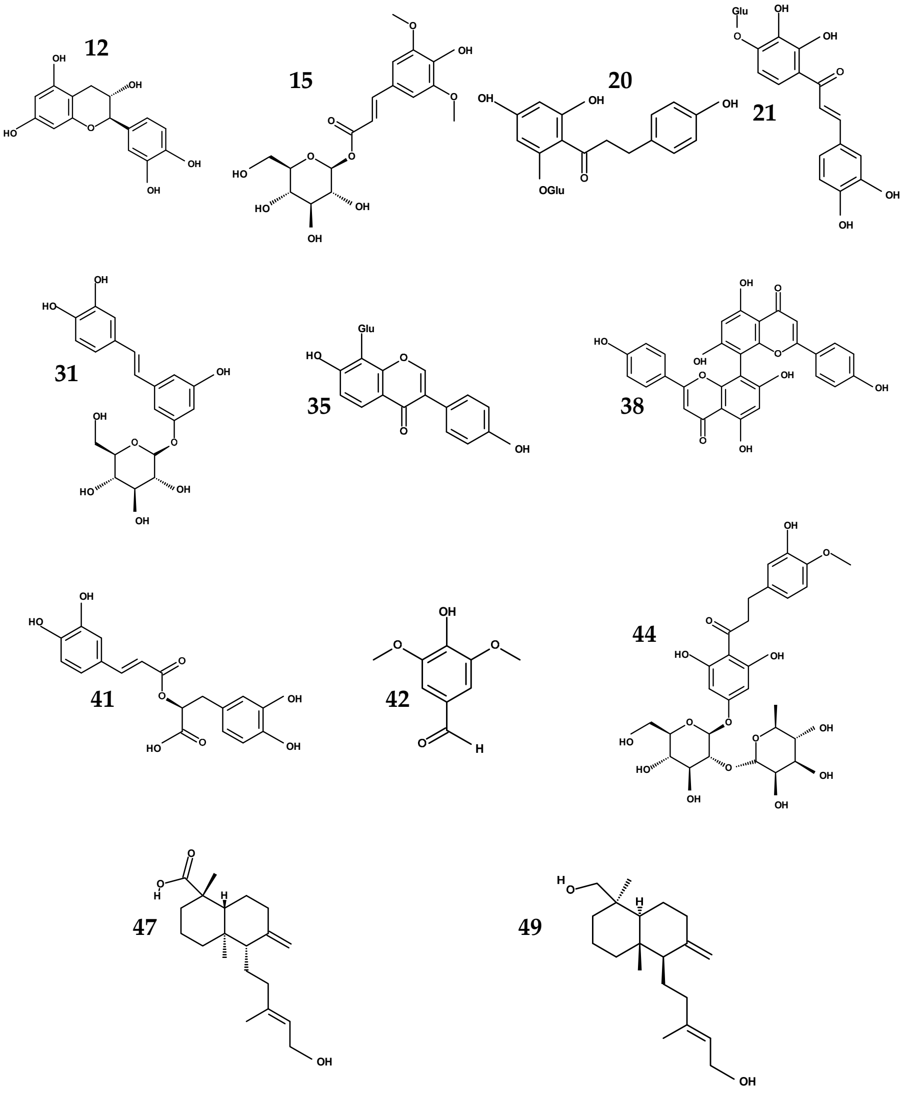

Compound No.	Compound name	Compound No.	Compound name
12	Catechin	38	Cupressuflavone
15	1-*O*-*β*-D-glucopyranosyl sinapate	41	Rosmarinic acid
20	Phlorizin	42	Syringaldehyde
21	Okanin-4'-*O*-glucoside	44	Neohesperidin dihydrochalcone
31	E-3,4,5'-Trihydroxy-3'-glucopyranosyl-stilbene	47	Isocupressic acid
35	Daidzein-8-*C*-glucoside	49	Agathadiol

Figure S3. Some compounds identified tentatively of *C. macrocarpa* leaves by LC-ESI-MS/MS (Glu=glucose)
